# Combined nutritional and inflammatory risk stratification for predicting functional outcomes in acute ischemic stroke

**DOI:** 10.3389/fnut.2026.1869783

**Published:** 2026-07-07

**Authors:** Baozhi Jia, Rongrong Ma, Panpan Zhu, Rufang Zhang, Shijun Li, Zhuangzhuang Jiang

**Affiliations:** 1 Department of Neurology, Tianxiang East Hospital, Yiwu, China; 2 Department of Neurology, Affiliated Dongyang Hospital of Wenzhou Medical University, Dongyang, China

**Keywords:** acute ischemic stroke, inflammation, malnutrition, prognosis, risk stratification

## Abstract

**Background:**

Inflammation and malnutrition are both closely associated with adverse outcomes after acute ischemic stroke (AIS), yet their combined effect and potential interaction remain insufficiently understood. This study aimed to evaluate the prognostic value of combined nutritional and inflammatory risk stratification based on nutritional risk screening-2002 (NRS-2002) and neutrophil-to-lymphocyte ratio (NLR), and to explore whether inflammatory burden mediates the relationship between nutritional status and functional outcomes.

**Methods:**

We retrospectively analyzed 1,167 consecutive patients with AIS admitted within 48 h of symptom onset. Nutritional status was assessed using NRS-2002, and inflammatory status was evaluated using admission NLR. Patients were categorized into four groups: low-Risk, inflammatory-risk, nutritional-risk, and dual-risk. The primary outcome was poor functional outcome at 3 months (modified Rankin Scale ≥3). Multivariable logistic regression models were used to examine associations. Predictive performance was evaluated using AUC, NRI, and IDI. Mediation analysis was conducted using NLR area under the curve (NLR-AUC) to assess the indirect effect of inflammation.

**Results:**

Compared with the low-risk group, the inflammatory-risk, nutritional-risk, and dual-risk groups were all independently associated with increased risk of poor functional outcomes (all *P* < 0.05). The dual-risk group showed the highest risk (fully adjusted OR = 3.59, *P* < 0.001). Nutritional–inflammatory risk stratification demonstrated superior predictive performance compared with traditional nutritional indices (all *P* < 0.05). Although it did not significantly improve AUC when added to the totaled health risks in vascular events (THRIVE) score, it significantly enhanced continuous NRI and IDI (both *P* < 0.05). Mediation analysis revealed that inflammatory burden partially mediated the association between malnutrition and poor outcomes in the basic model (mediation proportion: 8.73%), but this effect was attenuated after full adjustment.

**Conclusions:**

Combined assessment of nutritional and inflammatory status provides meaningful risk stratification for functional outcomes in AIS. Malnutrition and systemic inflammation exert a synergistic effect on post-stroke prognosis, while inflammation may partially mediate this relationship but is not independent of key clinical factors.

## Introduction

Acute ischemic stroke (AIS) is a leading cause of death and disability worldwide (1). In 2021, it accounted for 69.9 million prevalent cases, 7.8 million incident cases, 3.6 million deaths, and 70.4 million disability-adjusted life years globally (1). Although age-standardized rates have declined, the absolute burden continues to rise due to population aging. Moreover, the proportion of IS among all stroke subtypes has increased from 68.32% in 1990 to 73.33% in 2021, making it the predominant contributor to the global stroke burden (2).

Accumulating evidence highlights the critical role of inflammation in determining the prognosis of ischemic stroke. Elevated high-sensitivity C-reactive protein levels within 24–72 h of stroke onset have been associated with an increased risk of poor long-term functional outcomes (3). Moreover, composite inflammatory indices, including the systemic inflammatory response index, systemic immune-inflammatory index, and inflammatory prognostic index, can predict unfavorable outcomes at 3 months in patients undergoing intravenous thrombolysis (4). A higher inflammatory burden index is also associated with an increased risk of 90-day unfavorable outcomes in patients treated with endovascular therapy (5). Novel inflammatory markers are similarly linked to stroke prognosis, with serum cytokine analysis showing Interleukin-5 as an independent protective factor and Interleukin-6 as an independent risk predictor in AIS patients (6).

Nutritional status is another factor closely linked to stroke prognosis. Malnutrition, as defined by the internationally recognized global leadership initiative on malnutrition criteria, is associated with poorer functional recovery, less favorable discharge outcomes, and an increased risk of mortality in stroke patients (7). Biochemistry-based nutritional risk assessment tools, including the geriatric nutritional risk index, controlling nutritional status score, and prognostic nutritional index, have demonstrated that better nutritional status reliably predicts improved functional recovery, a lower incidence of post-stroke complications, and reduced mortality in older adults with ischemic stroke (8, 9).

Nutritional and inflammatory composite indices are widely used across various medical fields. The systemic immune-inflammatory index and prognostic nutritional index have been employed to evaluate prognosis in patients with severe community-acquired pneumonia (10). Similarly, the combined use of immune and inflammatory markers has been applied to assess prognosis in patients with respiratory and gastrointestinal malignancies (11, 12). Incorporating nutritional and inflammatory indices into clinical indicators has also been shown to improve the diagnostic accuracy for severe pulmonary tuberculosis (13). However, research on the application of combined nutritional and inflammatory indices in stroke remains limited. The nutritional risk screening-2002 (NRS-2002) score is a well-established and widely used nutritional screening tool, recommended by both the European society for parenteral and enteral nutrition and the Chinese guidelines for acute ischemic stroke for assessing patients' nutritional status (14). The neutrophil-to-lymphocyte ratio (NLR) serves as the basis for many composite inflammatory indices and has been shown to be closely associated with prognosis in ischemic stroke (15, 16). This study aims to use NRS-2002 and NLR for risk stratification, to investigate their relationship with stroke outcomes, and to evaluate their predictive performance. In addition, we examine whether inflammatory status acts as a mediator between nutritional status and prognosis in patients with ischemic stroke.

## Materials and methods

### Patients

In this study, we retrospectively analyzed consecutive ischemic stroke patients admitted to the Department of Neurology at Tianxiang East Hospital and the Affiliated Dongyang Hospital of Wenzhou Medical University between January 2024 and June 2025. The study was approved by the Ethics Committees of Tianxiang East Hospital and the Affiliated Dongyang Hospital of Wenzhou Medical University. Written informed consent was obtained from all participants or their legal guardians upon admission. The study was conducted in accordance with the Declaration of Helsinki. The inclusion criteria were as follows: (1) a confirmed diagnosis of ischemic stroke based on the 2023 Chinese Guidelines for the diagnosis and treatment of acute ischemic stroke; (2) age >18 years; and (3) admission within 48 h of symptom onset. The exclusion criteria were as follows: (1) presence of acute infection at admission; (2) malignancy, autoimmune diseases, or severe hepatic or renal dysfunction; (3) a pre-stroke modified Rankin Scale (mRS) score ≥ 3; and (4) incomplete clinical data or loss to follow-up. As this was a retrospective study, no formal sample size calculation was performed. All consecutive eligible patients during the study period were included.

### Data collection

Information on patients' demographics, vascular risk factors, comorbidities, treatment modalities, and laboratory results was obtained from electronic medical records. Patients treated with endovascular therapy, either alone or following intravenous thrombolysis (bridging therapy), were classified in the endovascular treatment group. Stroke etiology was determined for all patients using the TOAST classification based on clinical data (17). Hospital-acquired infection was defined as any infection developing 48 h or more after admission, including respiratory infections, pneumonia, and bloodstream infections. At 3 months ± 1 week after stroke onset, outcomes were evaluated via outpatient visits or telephone follow-up using the modified Rankin Scale (mRS) (18), with mRS ≥ 3 defined as a poor outcome.

### Risk stratification based on nutritional and inflammatory status

The NRS-2002 was applied by trained nurses to evaluate the nutritional status of patients upon admission. The NRS-2002 score ranges from 0 to 7 and includes three components: (1) disease severity (indicating increased nutritional needs), ranging from 0 to 3 based on comorbidities and medical history; (2) nutritional impairment, based on BMI, body weight, and food intake, with a score range of 0 to 3; and (3) age, with one point assigned for patients aged 70 years or older (14). Patients were classified into a high nutritional risk group (NRS-2002 ≥3) and a low nutritional risk group (NRS-2002 < 3). NLR was calculated from the first complete blood count performed upon admission by dividing the absolute neutrophil count by the absolute lymphocyte count (NLR = neutrophil count ÷ lymphocyte count; 15). For patients with a follow-up NLR measured 5 ± 1 days after admission, and without infection at the time of retesting, the cumulative inflammatory burden was quantified using the area under the curve of NLR (NLR-AUC), calculated by the trapezoidal method as NLR-AUC= (admission NLR + retest NLR) × days/2, with “days” representing the time interval between the two measurements. Receiver operating characteristic curve analysis based on admission NLR identified 3.6 as the optimal threshold for predicting 3-month unfavorable outcomes (mRS ≥ 3). Patients were classified into a high inflammatory status group (admission NLR ≥ 3.6) and a low inflammatory status group (admission NLR < 3.6). Based on the combination of nutritional and inflammatory status, patients were further stratified into four nutritional–inflammatory risk groups: low-risk group (low nutritional risk and low inflammatory status), inflammatory-risk group (low nutritional risk but high inflammatory status), nutritional-risk group (high nutritional risk but low inflammatory status), and dual-risk group (high nutritional risk and high inflammatory status).

### Statistical analysis

Continuous variables were presented as medians with interquartile ranges (IQRs), and categorical variables as counts and percentages. Comparisons of continuous variables among the four independent groups were performed using the Kruskal–Wallis *H* test, followed by pairwise comparisons with Bonferroni correction. Differences in categorical variables were assessed using Fisher's exact test or the Chi-square (*X*^2^) test, as appropriate. Four logistic regression models incorporating different covariates were constructed to examine the association between nutritional–inflammatory risk stratification and poor functional outcomes at 3 months. The predictive performance of nutritional–inflammatory risk stratification for poor outcomes in ischemic stroke was evaluated using the area under the receiver operating characteristic curve (AUC), net reclassification improvement (NRI), and integrated discrimination improvement (IDI). Subgroup analyses were performed to explore potential effect modifiers in the association between nutritional–inflammatory risk stratification and 3-month poor functional outcomes. NLR-AUC was used as the mediator in mediation analysis to evaluate whether inflammatory burden mediates the relationship between nutritional status and 3-month poor functional outcomes. Statistical significance was defined as a two-sided *P* < 0.05. All analyses were performed using SPSS version 26.0 and R version 4.1.1.

## Results

### Baseline characteristics by nutritional–inflammatory risk stratification

A total of 1,167 patients with acute ischemic stroke were retrospectively included and stratified into four groups according to nutritional and inflammatory status: low-risk (*n* = 605), inflammatory-risk(*n* = 203), nutritional-risk (*n* = 211), and dual-risk (*n* = 148), as detailed in Table 1. Age increased progressively across the four groups, with median values of 62.00, 67.00, 77.00, and 82.00 years, respectively, while the proportion of males was lowest in the nutritional-risk group. Patients in the inflammatory-risk and dual-risk groups were more likely to undergo reperfusion therapy (*P* < 0.001). Compared with the other groups, the dual-risk group had a lower proportion of small-artery occlusion and a higher proportion of cardioembolic stroke (*P* < 0.001). Moreover, the dual-risk group exhibited a higher incidence of hospital-acquired infections, greater stroke severity, and a significantly higher rate of poor functional outcomes (*P* < 0.001).

**Table 1:** Baseline characteristics of patients with acute ischemic stroke stratified by nutritional and inflammatory status.

Variables	Low-risk group (*N* = 605)	Inflammatory-risk group (*N* = 203)	Nutritional-risk group (*N* = 211)	Dual-risk group (N=148)	*P*
Demographic data					
Age (years), median (IQR)	62.00 (54.00,70.00)	67.00 (56.00,74.00) a	77.00 (73.00,83.00) ab	82.00 (76.00,87.00) ab	< 0.001
Sex, male, *n* (%)	400 (66.1) 24.65 (22.27,26.70)	143 (70.4)	111 (52.6) ab	86 (58.1)	< 0.001
BMI (kg/m^2^), Median (IQR)		24.17 (21.97,26.57)	22.86 (20.81,25.10) ab	22.88 (20.76,25.39) ab	< 0.001
Vascular risk factors, *n* (%)					
Hypertension	459 (75.9)	159 (78.3)	169 (80.1)	113 (76.4)	0.612
Diabetes mellitus	212 (35.0)	76 (37.4)	61 (28.9)	39 (26.4)	0.059
Ischemic heart disease	68 (11.2)	30 (14.8)	37 (17.5)	36 (24.3) a	< 0.001
History of stroke/TIA	86 (14.2)	31 (15.3)	31 (14.7)	27 (18.2)	0.673
Current smoke	210 (34.7)	56 (27.6)	25 (11.8) ab	15 (10.1) ab	< 0.001
Excess alcohol consumption	119 (19.7)	41 (20.2)	32 (15.2)	24 (16.2)	0.391
Laboratory data, median (IQR)					
Hb (g/L)	141.00 (129.00,151.00)	141.00 (130.00,152.00)	130.00 (115.00,138.00) ab	137.00 (123.00,148.00) abc	< 0.001
TC (mmol/L)	4.26 (3.58,5.03)	4.29 (3.62,4.93)	3.93 (3.33,4.71) ab	3.87 (3.34,4.50) ab	< 0.001
TG (mmol/L)	1.36 (0.97,1.91)	1.07 (0.81,1.54) a	0.98 (0.75,1.35) a	0.85 (0.61,1.21) abc	< 0.001
LDL (mmol/L)	2.35 (1.77,3.01)	2.32 (1.69,2.98)	2.02 (1.48,2.65) ab	1.90 (1.36,2.37) ab	< 0.001
HDL (mmol/L)	1.01 (0.86,1.22)	1.00 (0.86,1.14)	1.05 (0.89,1.30) b	1.01 (0.87,1.23)	0.034
Hemoglobin A1c (%)	6.00 (5.70,6.95)	6.00 (5.70,6.90)	5.90 (5.70,6.40)	5.80 (5.60,6.07) ab	< 0.001
Reperfusion therapy, *n* (%)					< 0.001
No reperfusion therapy	535 (88.4%)	149 (73.4%) a	183 (86.7%) b	109 (73.6%) ac	
Intravenous thrombolysis	58 (9.6%)	21 (10.3%) a	24 (11.4%) b	22 (14.9%) ac	
Endovascular treatment	12 (2.0%)	33 (16.3%) a	4 (1.9%) b	17 (11.5%) ac	
TOAST classification, *n* (%)					< 0.001
Small-vessel occlusion	338 (55.9)	71 (35.0) a	100 (47.4) ab	40 (27.0) abc	
Large-artery atherosclerosis	137 (22.6)	76 (37.4) a	43 (20.4) ab	44 (29.7) abc	
Cardioembolism	32 (5.3)	21 (10.3) a	25 (11.8) ab	33 (22.3) abc	
Other/undetermined	98 (16.2)	35 (17.2) a	43 (20.4) ab	31 (20.9) abc	
Clinical characteristics and outcomes					
Hospital-acquired infection, *n* (%)	28 (4.6)	34 (16.7) a	29 (13.7) a	55 (37.2) abc	< 0.001
Admission NIHSS score, median (IQR)	1.00 (1.00,3.00)	2.00 (1.00,6.00) a	2.00 (1.00,3.00) a	4.00 (2.00,12.00) abc	< 0.001
Admission NIHSS score >5, *n* (%)	34 (5.6%)	63 (31.0%) a	24 (11.4%) ab	66 (44.6%) ac	< 0.001
mRS≥3, *n* (%)	60 (9.9)	48 (23.6) a	40 (19.0) a	66 (44.6) abc	< 0.001

BMI, body mass index; TIA, transient ischemic attack; Hb, hemoglobin; TC, total cholesterol; TG, triglyceride; LDL, low-density lipoprotein cholesterol; HDL, high-density lipoprotein cholesterol; NIHSS, national institutes of health stroke scale; TOAST, trial of Org 10172 in acute stroke treatment; mRS, modified rankin scale; IQR, interquartile range.

a indicates significant difference compared with low-risk group; b indicates significant difference compared with inflammatory-risk group; c indicates significant difference compared with nutritional-risk group (Bonferroni-adjusted *p*-values for pairwise comparisons).

### Association between nutritional-inflammatory risk stratification and functional outcome

Table 2 presents the associations between nutritional–inflammatory risk stratification and 3-month poor functional outcomes in patients with acute ischemic stroke, as assessed using four logistic regression models with the low-risk group as the reference. The inflammatory-risk, nutritional-risk, and dual-risk groups were all significantly associated with an increased risk of poor outcomes across all models (all *P* < 0.05). With progressive adjustment for potential confounders, the odds ratios were attenuated but remained statistically significant. Notably, the dual-risk group consistently showed the highest risk across all models (crude OR = 7.31; fully adjusted OR = 3.59; both *P* < 0.001).

**Table 2:** Nutritional-inflammatory risk stratification and 3-month poor functional outcome in acute ischemic stroke.

NNRS	Model1		Model2		Model3		Model4	
	OR (95% CI)	*P*	OR (95% CI)	*P*	OR (95% CI)	*P*	OR (95% CI)	*P*
Low-risk	1.00 (Reference)		1.00 (Reference)		1.00 (Reference)		1.00 (Reference)	
Inflammatory-risk	2.81 (1.85–4.28)	**< 001**	3.00 (1.96–4.60)	**< 001**	1.80 (1.08–3.01)	**0.024**	1.78 (1.06–2.99)	**0.030**
Nutritional-risk	2.12 (1.37–3.28)	**< 001**	2.31 (1.40–3.80)	**0.001**	2.02 (1.15–3.54)	**0.014**	1.98 (1.12–3.51)	**0.018**
Dual-risk	7.31 (4.81–11.12)	**< 001**	8.46 (5.05–14.17)	**< 001**	3.81 (2.05–7.07)	**< 001**	3.59 (1.91–6.75)	**< 001**

Model 1: Crude model. Model 2: Adjusted for age, gender, smoking, hypertension, diabetes mellitus, coronary artery disease, history of stroke/TIA. Model 3: Adjusted for Model 2 covariates plus reperfusion therapy, TOAST classification, admission NIHSS, hospital-acquired infection. Model 4: Adjusted for Model 3 covariates plus total cholesterol, triglycerides, low-density lipoprotein cholesterol, high-density lipoprotein cholesterol, hemoglobin A1c. NNRS, nutritional-inflammatory risk stratification; TIA, transient ischemic attack; TOAST, trial of Org 10172 in acute stroke treatment; NIHSS, national institutes of health stroke scale; OR, odds ratio; CI, confidence interval. Bold values indicate statistical significance (*p* < 0.05).

### Incremental prognostic value of nutritional-inflammatory risk stratification

**Figure 1:**
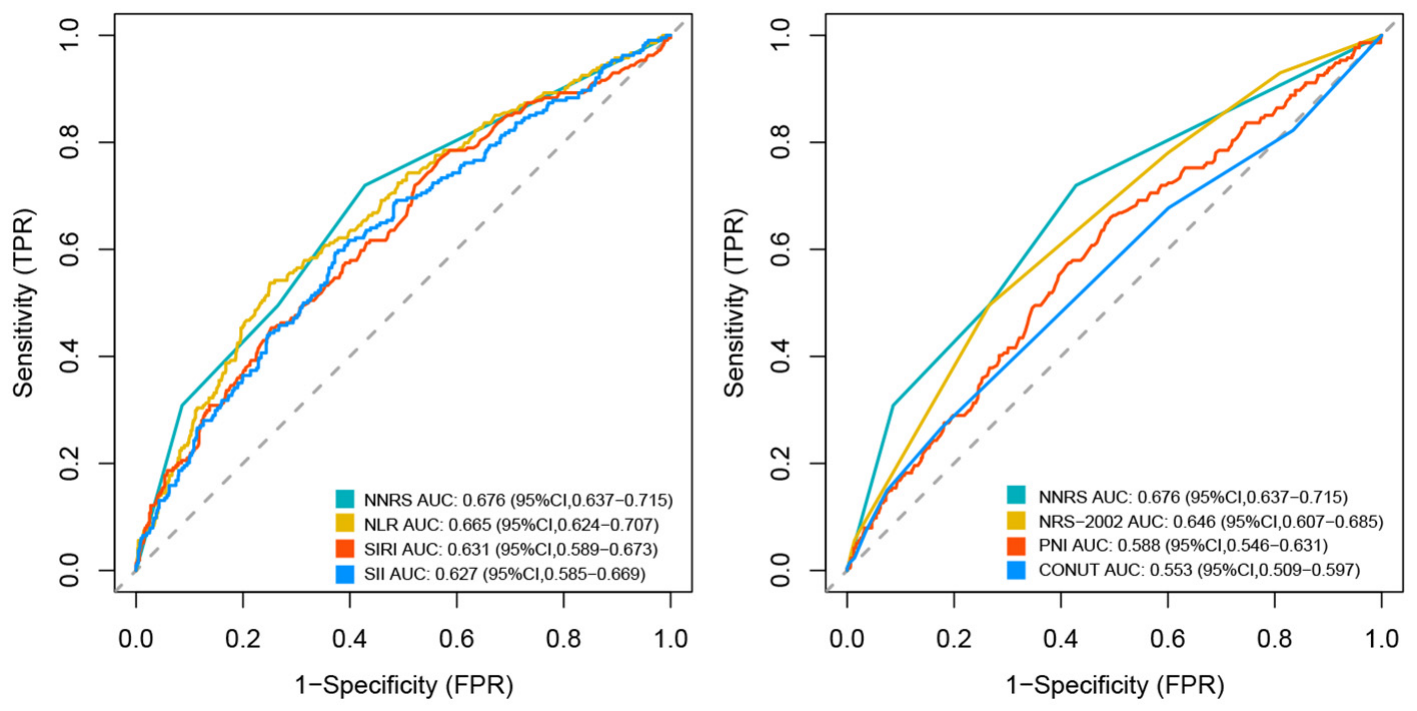
Comparison of area under the receiver operating characteristic curves (AUCs) for nutritional-inflammatory risk stratification (NNRS) and other inflammatory and nutritional markers in predicting poor outcome after ischemic stroke. The AUC of NNRS was significantly higher than those of SII, NRS-2002, PNI, and CONUT (DeLong test, *P* < 0.05), and higher than NLR and SIRI, but the differences were not statistically significant (DeLong test, *P* > 0.05). NRS-2002, Nutritional risk screening 2002; NLR, neutrophil-to-lymphocyte ratio (neutrophils ÷ lymphocytes); SIRI, systemic inflammatory response index (neutrophils × monocytes ÷ lymphocytes); SII, systemic immune-inflammation index (platelets × neutrophils ÷ lymphocytes); PNI, prognostic nutritional index [10 × albumin (g/Dl) + 0.005 × lymphocytes (per mm^3^)]; CONUT, controlling nutritional status score (based on serum albumin, lymphocyte count, and total cholesterol; each component scored 0–6 according to standard thresholds, summed to give total score).

As shown in Figure 1, nutritional-inflammatory risk stratification exhibited a higher AUC compared with traditional nutritional indices, including NRS-2002, PNI, and CONUT (all *P* < 0.05). For inflammatory markers, nutritional–inflammatory risk stratification showed a significantly greater AUC than SII (*P* < 0.05). Although its AUC was also higher than that of NLR and SIRI, these differences were not statistically significant (both *P* > 0.05). As presented in Table 3, the addition of nutritional–inflammatory risk stratification to the THRIVE score did not significantly improve AUC or categorical NRI (*P* > 0.05), but it significantly enhanced continuous NRI and IDI (both *P* < 0.05).

**Table 3:** Incremental prognostic value of nutritional–inflammatory risk stratification in acute ischemic stroke.

Predictive performance	THRIVE score	THRIVE score + NNRS	*P*
AUC (95% CI)	0.828 (0.796, 0.860)	0.830 (0.898, 0.862)	0.832
NRI^cat^ (95% CI)	Reference	−0.030 (−0.076, 0.016)	0.200
NRI^cont^ (95% CI)	Reference	0.217 (0.070, 0.365)	0.004
IDI (95% CI)	Reference	0.016 (0.006, 0.026)	0.001

AUC, area under the curve; CI, confidence interval; THRIVE, totaled health risks in vascular events; NRI^cat^, categorical net reclassification improvement; NRI^cont^, continuous net reclassification improvement; IDI, integrated discrimination improvement; NNRS, nutritional-inflammatory risk stratification.

THRIVE score: A validated prognostic scale for acute ischemic stroke based on age, baseline NIHSS, and the presence of hypertension, diabetes, and atrial fibrillation; higher scores indicate greater risk of poor outcomes.

### Subgroup analysis of nutritional–inflammatory risk stratification and poor functional outcomes

Subgroup analyses were conducted to examine the association between nutritional–inflammatory risk stratification and 3-month poor functional outcomes in patients with acute ischemic stroke across different clinical subgroups in Table 4. The association between nutritional–inflammatory risk stratification and poor prognosis was particularly pronounced in elderly patients and those with comorbidities such as hypertension and diabetes. A stronger relationship was also observed in patients with moderate-to-severe stroke and in those who did not undergo reperfusion therapy. Notably, the dual-risk group demonstrated a markedly increased risk of poor functional outcome in patients with large-artery atherosclerosis as well as in those with other or undetermined stroke etiologies. No significant interaction was observed (all *P* for interaction > 0.05).

**Table 4:** Subgroup analysis of nutritional–inflammatory risk stratification and 3-month poor outcomes (low-risk reference).

Subgroup	Inflammatory-risk group	Nutritional-risk group	Dual-risk group	*P* for interaction
Gender					0.822
Male (*N* = 740)	1.959 (1.049, 3.658)^*^	2.695 (1.227, 5.921)^*^	4.987 (2.224, 11.182)^***^	
Female (*N* = 427)	1.218 (0.458, 3.240)	2.209 (0.935, 5.217)	3.970 (1.551, 10.163)^**^	
Age					0.291
≥65 (*N* = 718)	1.928 (0.915, 4.065)	2.284 (1.192, 4.374)^*^	4.815 (2.429, 9.545)^***^	
< 65 (*N* = 449)	1.161 (0.509, 2.649)	15.388 (1.221, 193.920)^*^	3.401 (0.128, 90.220)	
Hypertension					0.439
Yes (*N* = 900)	1.789 (0.984, 3.254)	2.384 (1.240, 4.582)^**^	5.255 (2.628, 10.508)^***^	
No (*N* = 267)	1.322 (0.397,4.401)	3.052 (0.898, 10.372)	4.570 (1.181, 17.683)^*^	
Diabetes mellitus					0.697
Yes (*N* = 388)	2.062 (0.894, 4.755)	3.690 (1.447, 9.411)^**^	7.015 (2.469, 19.927)^***^	
No (*N* = 779)	1.503 (0.761, 2.967)	1.879 (0.901, 3.919)	3.550 (1.662, 7.584)^*^	
NIHSS score					0.957
>5 (*N* = 187)	4.846 (1.537, 15.277)^**^	6.464 (1.497, 27.924)^*^	18.255 (4.400, 75.742)^***^	
≤ 5 (*N* = 980)	1.518 (0.791, 2.910)	2.026 (1.073, 3.827)^*^	3.910 (1.900, 8.047)^***^	
TOAST classification					0.101
SVO (*N* = 549)	1.141 (0.482, 2.702)	2.512 (1.097, 5.749)^*^	2.821 (0.969, 8.208)	
LAA (*N* = 300)	3.603 (1.345, 9.654)^*^	1.692 (0.504, 5.685)	8.375 (2.530, 27.718)^**^	
CE (*N* = 111)	0.261 (0.016, 4.202)	0.419 (0.039, 4.514)	2.306 (0.257, 20.688)	
OU (N=207)	13.109 (1.221, 140.709)^*^	35.090 (2.137, 576.073)^*^	63.538 (3.677, 1097.795)^**^	
Reperfusion therapy					0.08
No (*N* = 976)	1.914 (1.047, 3.500)^*^	2.083 (1.098, 3.951)^*^	4.443 (2.232, 8.844)^***^	
Intravenous thrombolysis (*N* = 125)	0.820 (0.191, 3.510)	2.898 (0.613, 13.699)	1.508 (0.288, 7.890)	

NNRS, nutritional-inflammatory risk stratification; NIHSS, national institutes of health stroke scale; TOAST, trial of Org 10172 in acute stroke treatment; SVO, small-vessel occlusion; LAA, large-artery atherosclerosis; CE, Cardioembolism; OU, Other/undetermined; N, Number. Adjusted for covariates in Model 4. Statistical significance is denoted as: ^*^ *p* < 0.05, ^**^ *p* < 0.01, ^***^ *p* < 0.001.

### Mediating role of inflammation in nutrition and functional outcomes

**Figure 2:**
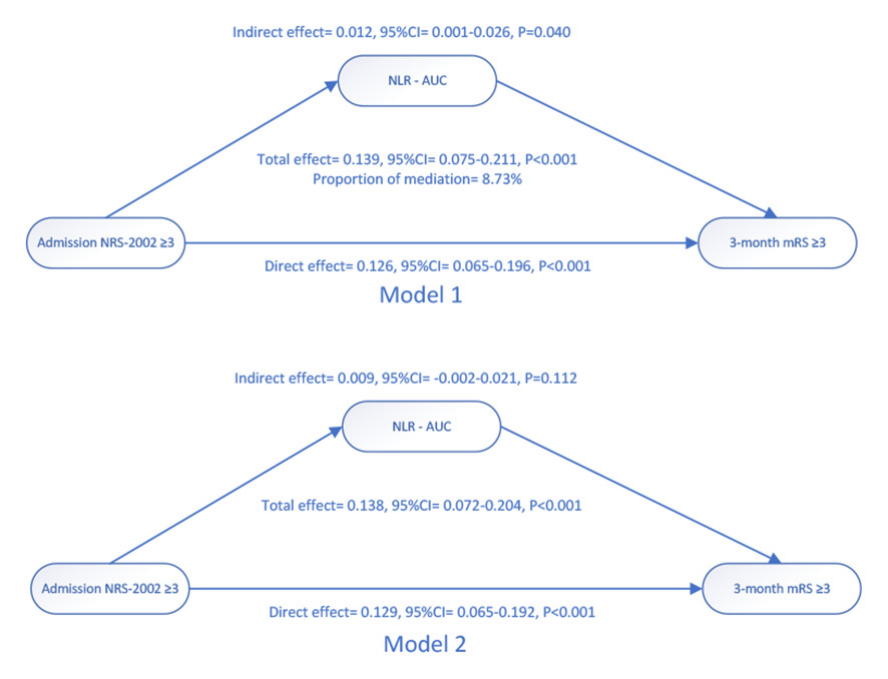
Mediating effect of inflammation burden, measured by neutrophil-to-lymphocyte ratio area under the curve (NLR-AUC; mediator), on the association between malnutrition (nutritional risk screening 2002 ≥3; exposure) and poor 3-month functional outcome (modified Rankin scale ≥3; outcome) in patients with acute ischemic stroke. NLR-AUC partially mediated this association in Model 1 (accounting for 8.73% of the total effect), whereas the mediating effect was attenuated and became non-significant in Model 2. Model 1: adjusted for age, sex, cigarette smoking, hypertension, diabetes mellitus, coronary artery disease, and history of stroke or transient ischemic attack. Model 2: adjusted for all Model 1 covariates plus reperfusion therapy, Trial of Org 10172 in Acute Stroke Treatment classification, admission National Institutes of Health Stroke Scale score, hospital-acquired infection, total cholesterol, triglycerides, low-density lipoprotein cholesterol, high-density lipoprotein cholesterol, and glycated hemoglobin.

Mediation analyses were conducted to evaluate whether inflammatory status mediates the association between nutritional status and prognosis in patients with acute ischemic stroke in Figure 2. In Model 1, NLR-AUC demonstrated a significant partial mediating effect (indirect effect = 0.012, 95% CI: 0.001–0.026, *P* = 0.040), accounting for 8.73% of the total effect of NRS-2002 on 3-month mRS. In Model 2, the mediating effect of NLR-AUC was attenuated and no longer statistically significant (indirect effect = 0.009, 95% CI: −0.002 to 0.021, *P* = 0.112).

## Discussion

After ischemic stroke, hypoxia and ischemia cause neuronal necrosis and the release of damage-associated molecular patterns, which activate microglia and trigger central neuroinflammation (19). This response leads to excessive production of pro-inflammatory cytokines (e.g., TNF-α, IL-1β, and IL-6), disruption of the blood–brain barrier, and infiltration of peripheral immune cells, especially neutrophils, into the brain, further worsening neuronal injury and promoting the progression of the ischemic penumbra to irreversible infarction (19, 20). Overactivation of central immune responses also disrupts peripheral immunity, resulting in immunosuppression that increases susceptibility to infections (20). At the same time, malnutrition is common in the acute phase of stroke, with reported prevalence ranging from 6.1% to 62%, and elderly patients being particularly vulnerable (21). Malnutrition reduces protein synthesis and glucose utilization in the ischemic penumbra, limiting neuronal repair (22), while also weakening immune defenses, increasing the risk of complications such as stroke-associated pneumonia and gastrointestinal bleeding (23), and contributing to muscle weakness that impairs rehabilitation and functional recovery (24). Beyond its impact on the acute phase, malnutrition may also adversely affect long-term recovery through persistent energy deficiency and the development of post-stroke sarcopenia (25). Inadequate nutritional intake promotes muscle protein breakdown and loss of muscle mass and strength, which can reduce physical performance, limit participation in rehabilitation programs, and ultimately impair functional recovery (26). Furthermore, chronic inflammation may exacerbate skeletal muscle wasting and cachexia through persistent catabolic signaling, creating a vicious cycle between malnutrition, inflammation, and sarcopenia that contributes to long-term disability after stroke (27). Consistent with these mechanisms, our study showed that patients with both high nutritional risk and high inflammatory status had the highest rate of poor functional outcomes at 3 months. To our knowledge, this is the first study to combine nutritional and immune–inflammatory indicators for risk stratification and prognosis prediction in ischemic stroke, highlighting the synergistic impact of malnutrition and systemic inflammation on post-stroke recovery.

In our study, comparison between the inflammatory-risk group and the nutritional-risk group revealed distinct patterns of inflammatory and nutritional risk among different stroke populations. Patients in the nutritional-risk group were older, had a higher proportion of females, and lower lipid levels, reflecting not only decline in nutritional reserves and muscle mass but also potential differences in metabolic and hormonal status that predispose them to malnutrition (28). By comparison, the inflammatory-risk group had a higher proportion of large-artery atherosclerotic strokes and a lower proportion of small-artery occlusion strokes, with higher baseline NIHSS scores, as large-artery atherosclerotic infarctions generally involve a larger infarct volume than small-artery occlusions (29), eliciting a more pronounced systemic immune–inflammatory response and resulting in more severe neurological deficits (30, 31). Notably, the dual-risk group had a higher proportion of cardioembolic strokes, which are typically sudden in onset and lack effective collateral circulation (32), leading to larger infarct volumes and more pronounced systemic inflammatory responses. Moreover, these patients often present with impaired consciousness or dysphagia in the acute phase, limiting energy and nutrient intake and thereby increasing the risk of malnutrition (33). The incidence of hospital-acquired infections was higher in all three risk groups compared with the low-risk group, indirectly indicating that impaired immune–inflammatory status and poor nutritional status may increase the risk of poor outcomes by promoting in-hospital complications.

Logistic regression analysis demonstrated that combined immune–inflammatory and nutritional risk stratification was more strongly associated with poor post-stroke outcomes than either inflammatory or nutritional indicators alone, and this association remained robust after adjustment for multiple potential confounders. Subgroup analyses further showed that this association was broadly consistent across patient subgroups, with no significant interactions observed. Notably, a trend toward stronger associations was evident in high-risk populations, including elderly patients, those with hypertension or diabetes, and patients with moderate-to-severe stroke. These populations are typically characterized by diminished physiological and nutritional reserves alongside heightened immune–inflammatory responses (28, 31), potentially predisposing them to worse outcomes. Furthermore, the association appeared more pronounced in patients without reperfusion therapy and in certain stroke subtypes, such as large-artery atherosclerosis, possibly reflecting both prolonged ischemic exposure and sustained systemic inflammatory responses in the absence of timely reperfusion (31), as well as the larger infarct burden and more extensive inflammatory activation typically observed in these conditions (30), which together may amplify the detrimental effects of nutritional and inflammatory disturbances.

Nevertheless, nutritional-inflammatory risk stratification did not improve the predictive AUC compared with NLR, and its overall discriminative ability remained modest. This may be attributable to the heterogeneity of the study population, as patients across a wide age range and receiving different treatment modalities were included, potentially diluting its predictive performance. The THRIVE score, a validated prognostic tool incorporating age, baseline NIHSS score, and key vascular comorbidities (34), demonstrated strong baseline predictive ability. The addition of nutritional-inflammatory risk stratification to the THRIVE score did not improve AUC or categorical NRI, likely because age and NIHSS already capture substantial prognostic information and may partially overlap with nutritional-inflammatory risk stratification. However, nutritional-inflammatory risk stratification significantly improved continuous NRI and IDI, suggesting that it provides incremental predictive value and enhances individual-level risk stratification beyond that achieved by the THRIVE score alone.

Previous studies have suggested that malnutrition may exacerbate post-stroke immune–inflammatory responses through impaired immune regulation, negative energy balance, and barrier dysfunction, leading to a pro-inflammatory and immunosuppressed state (35, 36). Based on this rationale, we conducted a mediation analysis to examine whether inflammation contributes to the association between malnutrition and stroke outcomes. Considering that post-stroke immune–inflammatory responses evolve dynamically and typically plateau within the first week (19), we used inflammatory burden (NLR-AUC) rather than admission NLR to capture the cumulative response. NLR-AUC partially mediated the association between malnutrition and poor functional outcomes in Model 1, accounting for 8.73% of the total effect, indicating that systemic inflammation is one pathway linking malnutrition to adverse prognosis. After further adjustment for clinical severity, stroke characteristics, and in-hospital factors in Model 2, the mediation effect was attenuated and no longer significant, suggesting that the observed mediation may be partly explained by factors closely related to both inflammation and outcomes, particularly stroke severity. These findings indicate that while inflammation may partially link malnutrition to poor outcomes, its effect is not independent of key clinical determinants, highlighting the complex interplay among nutritional status, systemic inflammation, and stroke severity in post-stroke recovery.

This study has several limitations. First, as a single-center retrospective study, it may be subject to selection bias and limit the generalizability of our findings. Second, patients with malignancy, autoimmune diseases, or severe hepatic or renal dysfunction were excluded to minimize their impact on inflammatory markers, but this may have overlooked their potential effects on nutritional status. Finally, although inflammatory burden was included in the mediation analysis, dynamic changes in nutritional status during hospitalization were not captured.

## Conclusion

In conclusion, our study demonstrates that combined assessment of nutritional and inflammatory status using NRS-2002 and NLR provides meaningful risk stratification for functional outcomes in patients with acute ischemic stroke. Patients with both high nutritional risk and high inflammatory burden exhibited the poorest prognosis, supporting a synergistic effect of malnutrition and systemic inflammation on post-stroke recovery. While inflammation may partially mediate this association, its effect is not independent of key clinical factors.

## Data Availability

The raw data supporting the conclusions of this article will be made available by the authors, without undue reservation.
